# Brachial-ankle pulse wave velocity is associated with the risk of osteoporosis: a cross-sectional evidence from a Chinese community-based cohort

**DOI:** 10.1186/s13018-020-02125-3

**Published:** 2021-01-04

**Authors:** Kun Tang, Qiao Zhang, Nianchun Peng, Ying Hu, Shujing Xu, Miao Zhang, Rui Wang, Lixin Shi

**Affiliations:** 1 Department of Endocrinology, The Second Affiliated Hospital of Soochow University, Suzhou, 215004 China; 2 Department of Endocrinology, The Affiliated Hospital of Guizhou Medical University, Guiyang, 550000 China

**Keywords:** Brachial-ankle pulse wave velocity (baPWV), Osteoporosis Self-Assessment Tool for Asia (OSTA), Osteoporosis, Arterial stiffness

## Abstract

**Background:**

Association of arterial stiffness and osteoporosis has been well documented in elderly population. However, it is not clear whether they co-progress from the early stages through common mechanisms. The object of this study was to evaluate possible associations between arterial stiffness and osteoporosis by measuring brachial-ankle pulse wave velocity (baPWV) and the Osteoporosis Self-Assessment Tool for Asia (OSTA) index among a healthy population of Chinese aged 40 years and older. Whether baPWV can be used as a predictor of osteoporosis on OSTA was further assessed.

**Methods:**

This study was cross-sectional in design. Of 3984 adults aged 40 years and older in the Yunyan district of Guiyang (Guizhou, China) who underwent both OSTA and baPWV measurements within 1 month, 1407 were deemed eligible for inclusion (women, 1088; men, 319).

**Results:**

The mean baPWV was 1475 ± 302 cm/s (range,766–3459 cm/s). baPWV in 110 individuals with high risk of osteoporosis (OSTA index < − 4) was higher than that of individuals with non-high risk (1733 ± 461 cm/s vs. 1447 ± 304 cm/s, *P* < 0.001). OSTA index was negatively correlated with baPWV(*ρ* = − 0.296, *P* < 0.001) after adjusting for age, sex, body mass index, waist circumference, diastolic blood pressure, and creatinine clearance rate. baPWV was an independent predictor for the presence of high risk of osteoporosis (*β* = − 0.001, *P* < 0.001) and was consistent across age and sex subgroups, and the optimal baPWV cutoff value for predicting the presence of high risk of osteoporosis and fracture was 1693 cm/s. The AUC was 0.722 (95% confidence interval [CI], 0.667–0.777; *P* < 0.001, sensitivity of 52.8% and specificity of 83.6%).

**Conclusions:**

We conclude that arterial stiffness measured by baPWV is well correlated with the severity of osteoporosis evaluated by OSTA. baPWV index may be a valuable tool for identifying individuals with risk of developing osteoporosis.

**Supplementary Information:**

The online version contains supplementary material available at 10.1186/s13018-020-02125-3.

## Background

Osteoporosis is a systemic skeletal condition of low bone mass resulting in micro-architectural deterioration of bone tissue, leaving the bones brittle and prone to fractures [1]. According to the latest nationwide, multicenter survey in China, a total of 60 million individuals (10.9 million men and 49.3 million women) are estimated to have osteoporosis [2]. Arterial stiffness, also known as atherosclerosis, is a phenomenon that results with an increase of vascular stiffness loss of elasticity, vessel wall calcification, and blood flow restriction affecting the media of large- and middle-sized arteries [3]. Both arterial stiffness and osteoporosis share some common risk factors and clinical characteristics [4–6].

Decreased bone mineralization (osteoporosis) often occurs concurrently with increased vascular calcification (arterial stiffness), probably due to the common osteogenic and mineralization process shared by bone and vascular cells [7]. Several clinical findings in the past have supported the correlation of osteoporosis or low bone mineral density (BMD) and arterial stiffness, indicating the parallel progression of the two chronic diseases with increased cardiovascular events and higher fracture risk [8–11].

According to the World Health Organization criteria, the gold standard method to assess and diagnose osteoporosis is based on dual-energy X-ray absorptiometry [12]. However, this tool also has certain limitations (e.g., high costs and large size of the equipment, exposure to ionizing radiation) that limit its widespread application for population screenings. On the other hand, some elderly and high-risk individuals often do not undergo regular screening and comprehensive examinations, probably due to health or financial reasons [13]. Among several currently used tools [14–16], the Osteoporosis Self-Assessment Tool for Asians (OSTA) score developed by WHO has been shown to be the simplest and highly effective tool to identify both women and men at risk for osteoporosis [6, 17]. It has been concluded that the OSTA was a useful screening tool to detect osteoporosis in middle-aged and old women in the Chengdu region of China [18]. A significant correlation in a positive direction was found between the OSTA index and BMD (*T* score) measured by dual-energy X-ray absorptiometry [19, 20]. Brachial-ankle pulse wave velocity (baPWV), recording the time taken by the pressure wave to travel over a specific distance, is the most common measure of arterial stiffness in Asian populations and is widely used as it is convenient, noninvasive, and inexpensive [21–23].

The measurement of arterial stiffness of healthy adults undergoing screening medical examination might help to make decisions on early intervention for osteoporosis or facture risk, since correlation of arterial stiffness by measuring baPWV and osteoporosis (BMD or other assessment indices) has been reported in recent studies [24, 25]. Xuan et al.’s work was the first to investigate the correlation between arterial stiffness (baPWV) and OSTA index in Chinese population. However, their findings were based on 129 elderly Chinese community-dwelling individuals with mean age above 83.2; it is not clear whether the one was the result of the other or they co-progress from the early stages through common mechanisms. Therefore, the aim of this study was (1) to investigate the relation between baPWV measurement and the OSTA index and (2) to evaluate baPWV as a predictor of risk of osteoporosis in 3984 Chinese healthy adults aged 40 years and older.

## Methods

### Study population

**Fig. 1:**
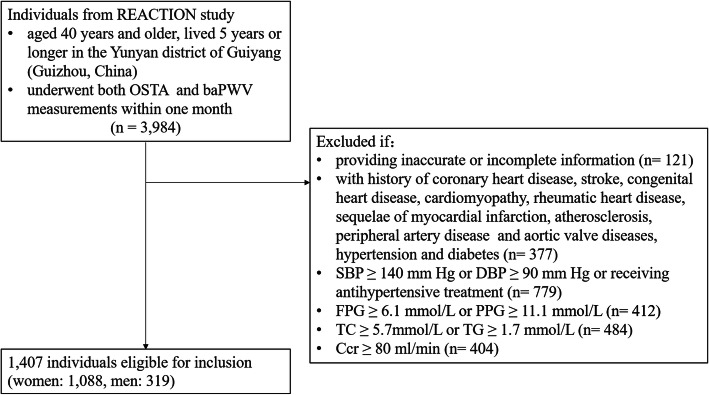
Workflow of sample enrollment of this study

The present study was part of the baseline survey for the Risk Evaluation of Cancers in Chinese Diabetic Individuals: a longitudinal (REACTION) study, which was conducted among 259,657 adults aged 40 years and older in 25 communities across mainland China from 2011 to 2012 [26–29]. The study protocol was approved by the ethics committees at Guizhou Medical University Affiliated Hospital and informed consent had been obtained from each participant who signed a form before the study. A total of 3984 adults aged 40 to 80 years old who lived 5 years or longer in the Yunyan district of Guiyang (Guizhou, China) were selected by cluster random sampling. Major exclusion criteria are shown in Fig. 1 and 1407 individuals were deemed eligible for inclusion (women, 1088; men, 319).

### Study method

1. Questionnaire survey: Information collected includes demographic and other background information (sex, age, occupation, home address, contact number, family history, marital and childbirth history, etc.) and past medical history (history of diabetes, hypertension, dyslipidemia, cardiovascular disease history, etc. and treatment of related diseases).
2. Physical examination: Height, body weight, waist circumference (WC), and blood pressure (BP) were measured. BP was measured three times in the supine position and the average was taken as the measurement value. The body mass index (BMI = weight/height^2^) was calculated.
3. Sample collection and measurement: Venous blood samples were obtained in the morning and an overnight fasting. Fasting plasma glucose (FPG) concentration and postprandial glucose (PPG) levels were measured using the glucose oxidase technique with a Roche Hitachi P800 autoanalyzer (Roche Diagnostics GmbH, Mannheim). Lipid panel test including total cholesterol (TC), high-density lipoprotein cholesterol (HDL), low-density lipoprotein cholesterol (LDL), and triglyceride (TG) was measured using an autoanalyzer (ARCHITECT Ci16200). We used creatinine clearance rate (Ccr) as an indicator of kidney function.
4. baPWV measurement: After a 10-min rest in the supine position, baPWV was automatically measured using the Omron device (BP-203RPEIII VP-1000 device; Omron Health Care, Kyoto, Japan) by a single observer with high level of operator experience. The validity and reliability of this device has been previously reported [30]. Cuffs with sensors were wrapped on both upper arms and ankles. The transmission times and distances between the cuffs of arms and legs were recorded, and the device was able to compute baPWV automatically as the ratio of the travel distance divided by the time difference between the pulse waves. We used the mean value of the right and left baPWVs in our analysis [31, 32].
5. OSTA index calculation: OSTA index was calculated using the formula of (weight in kilograms – age in years) × 0.2, established by Koh et al. (2001) [17]. An OSTA index of − 1 to − 4 is regarded as medium risk of sustaining osteoporosis, greater than − 1 as low risk, and less than − 4 as high risk [33, 34].
6. Subgroup stratification: The study population was then divided into subgroups according to sex and age (< 65 vs. ≥ 65 years old) for analysis, considering osteoporosis starts earlier and gets worse faster in women; however, starting at about age 65, both sexes lose bone at about the same rate [35, 36].

### Statistical analyses

Continuous variables were expressed as means ± standard deviation (SD). For assessment of the differences in a variable between 2 groups, the unpaired *t* test was applied. Spearman’s rank correlation analyses and partial correlation analyses were conducted to study the associations between baPWV and OSTA, anthropometric indices, and serum biochemical parameters. Multivariable regression analysis was performed to assess whether baPWV was independently associated with osteoporosis and using variables showing significant relationships with OSTA in Spearman’s rank correlation analysis. To explore the best cutoff value of baPWV for predicting high risk of osteoporosis and fracture (OSTA ≤ − 4), receiver operating characteristic (ROC) curve analysis was used. The predictive accuracy was presently with the area under the curve (AUC). Youden index, which was sensitivity + specificity – 1, was calculated to enable the selection of an optimal threshold value (cutoff point) [37]. The statistical analysis was performed using SPSS 19.0 software (SPSS, Inc., Chicago, IL, USA).

## Results

### Baseline characteristics

The baseline anthropometric parameters and biochemical indices from 1407 individuals are shown in Table 1. Males (age, 62.33 ± 7.99 years) were significantly older than females (age, 58.14 ± 7.52 years). Females had higher HDL, TC, and Ccr (*P* < 0.05). BMI, SBP, DBP, and PPG were higher in men than in women (all *P* < 0.05). For total individuals, the mean baPWV was 1475 ± 302 cm/s (range, 766–3,459 cm/s) and there was no significant difference between males and females in baPWV. Risk of osteoporosis measured by OSTA index is significantly higher in females (0.34 ± 2.53 in males vs. − 0.73 ± 2.30 in females).

**Table 1:** General characteristics of male and female patients

*N*	Male	Female	*T* value	*P* value
	319	1088		
Age (year)	62.33 ± 7.99	58.14 ± 7.52	8.347	< 0.001
BMI (kg/m^2^)	23.04 ± 2.74	22.54 ± 2.89	2.789	0.005
WC (cm)	85.14 ± 7.72	84.16 ± 8.57	1.832	0.067
SBP (mmHg)	118.89 ± 11.23	113.77 ± 12.37	6.648	< 0.001
DBP (mmHg)	76.22 ± 7.66	73.00 ± 7.99	6.401	< 0.001
FPG (mmol/L)	5.28 ± 0.47	5.26 ± 0.40	0.513	0.608
PPG (mmol/L)	7.02 ± 1.62	6.79 ± 1.52	2.276	0.023
HDL (mmol/L)	1.44 ± 0.28	1.49 ± 0.28	2.440	0.015
LDL (mmol/L)	2.73 ± 0.57	2.78 ± 0.52	1.450	0.147
TC (mmol/L)	4.72 ± 0.62	4.82 ± 0.58	2.472	0.014
TG (mmol/L)	1.14 ± 0.30	1.11 ± 0.31	1.508	0.132
Ccr (mL/min)	89.23 ± 8.77	92.68 ± 11.13	5.789	< 0.001
baPWV (cm/s)	1461.43 ± 391.11	1471.55 ± 307.43	0.425	0.671
OSTA	0.34 ± 2.53	− 0.73 ± 2.30	6.813	< 0.001

*BMI* body mass index, *WC* waist circumstances, *SBP*, systolic blood pressure, *DBP* diastolic blood pressure, *FPG* fasting plasma glucose, *PPG* postprandial glucose, *HDL* high-density lipoprotein cholesterol, *LDL* low-density lipoprotein cholesterol, *TC* total cholesterol, *TG* triglyceride, *Ccr* creatinine clearance, *baPWV* brachial-ankle pulse wave velocity, *OSTA* Osteoporosis Self-Assessment Tool for Asia

### Association of OSTA with baPWV and biochemical parameters

**Fig. 2:**
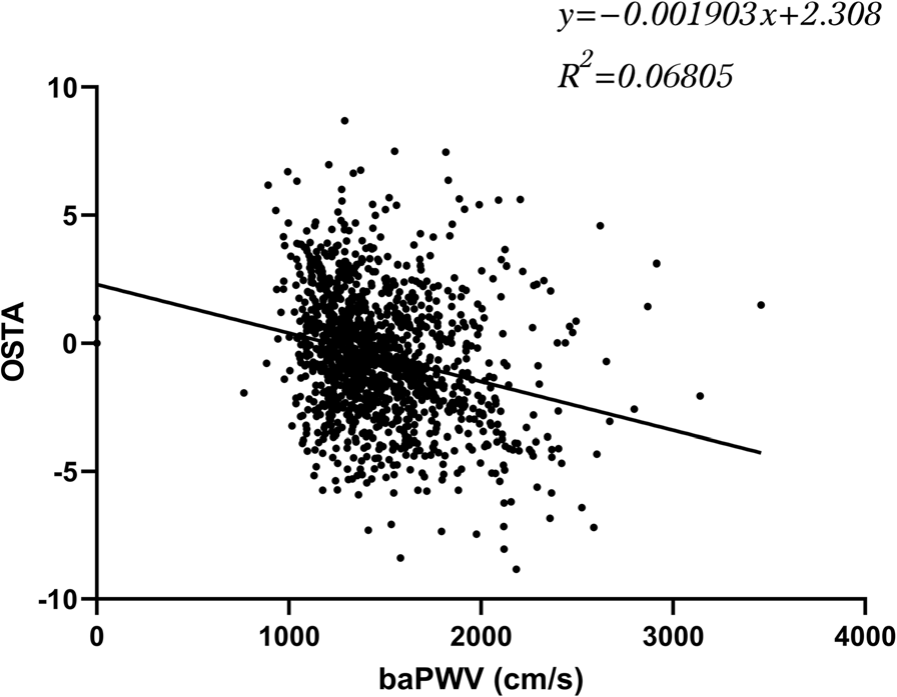
Scatter plot showing the negative association between OSTA index and baPWV. baPWV, brachial-ankle pulse wave velocity; OSTA, Osteoporosis Self-Assessment Tool for Asia

Spearman’s rank correlation analysis presented a significant negative correlation between OSTA index and baPWV (ρ = − 0.290, P < 0.001) (Table 2; Fig. 2). Further, we calculated the partial correlation between OSTA index and baPWV, adjusting for age, sex, BMI, WC, DBP, and Ccr. The correlation in a negative direction between OSTA index and baPWV was not significantly affected by other demographic or biochemical variables (ρ = − 0.294, P < 0.001).

**Table 2:** Correlations of OSTA index with baPWV and various clinical and biochemical parameters

	OSTA index	
	*ρ*	*P* value
baPWV	− 0.290	< 0.001
Age	− 0.543	< 0.001
Sex	− 0.174	< 0.001
BMI	0.459	< 0.001
WC	0.380	< 0.001
FPG	0.000	0.993
PPG	− 0.036	0.172
SBP	0.000	0.996
DBP	0.182	< 0.001
Ccr	0.239	< 0.001
HDL	− 0.042	0.113
LDL	0.009	0.724
TC	− 0.022	0.402
TG	0.024	0.370

*BMI* body mass index, *WC* waist circumstances, *SBP* systolic blood pressure, *DBP* diastolic blood pressure, *FPG* fasting plasma glucose, *PPG* postprandial glucose, *HDL* high-density lipoprotein cholesterol, *LDL* low-density lipoprotein cholesterol, *TC* total cholesterol, *TG* triglyceride, *Ccr* creatinine clearance, *baPWV* brachial-ankle pulse wave velocity, *OSTA* Osteoporosis Self-Assessment Tool for Asia

### baPWV as a predictor of high risk of osteoporosis

Stepwise multiple regression analysis was further applied to access the independent relationships between OSTA and baPWV. As shown in Table 3, multivariate stepwise regression analysis revealed that among those factors that showed associations with OSTA in correlation analysis, baPWV was an independent factor responsible for the changes in OSTA (β = − 0.001, P < 0.001). The regression equation was OSTA = 5.317 + 0.279 × BMI − 0.182 × Age − 1.642 × Sex + 0.042 × WC − 0.001 × baPWV.

**Table 3:** Multivariate stepwise regression analysis showing the factors independently associated with OSTA

Characteristics	*β*	SE	Standardized *β*	*P*
BMI	0.279	0.021	0.334	< 0.001
Age	− 0.182	0.005	− 0.594	< 0.001
Sex	− 1.642	0.093	− 0.287	< 0.001
WC	0.042	0.007	0.146	< 0.001
baPWV	− 0.001	0.000	− 0.185	< 0.001

Note: Multivariable stepwise regression analysis was performed using variables showing significant relationships with OSTA in Spearman’s rank correlation analysis

*BMI* body mass index, *WC* waist circumstances, *Ccr* creatinine clearance, *baPWV* brachial-ankle pulse wave velocity, *OSTA* Osteoporosis Self-Assessment Tool for Asia

Overall, 110 individuals with high risk of osteoporosis (OSTA index < − 4) was identified; baPWV measurement of these individuals was higher than that of individuals with non-high risk (1733 ± 461 cm/s vs. 1447 ± 304 cm/s, *P* < 0.001). In the ROC curve analysis, the AUC was 0.722 (95% confidence interval [CI], 0.667–0.777; *P* < 0.001), and the optimal cutoff point of baPWV value for predicting the presence of high risk of osteoporosis and fracture was 1693 cm/s with sensitivity of 52.8% and specificity of 83.6% (Supplementary file 1).

### Age and sex subgroup analysis

Further subgroup analysis showed that baPWV was an independent factor responsible for the changes in OSTA in each subgroup (Table 4). In male population, the AUC was 0.716 (95% confidence interval [CI], 0.545–0.886; *P* = 0.005), slightly lower than the AUC in female population (AUC 0.726; 95% confidence interval [CI], 0.669–0.783; *P* < 0.001). In individuals with age under 65, the AUC was 0.789 (95% confidence interval [CI], 0.650 to 0.929; *P* < 0.001), much higher than the AUC in individuals with age above 65 (AUC 0.662; 95% confidence interval [CI], 0.595 to 0.729; *P* < 0.001) (Fig. 2).

**Table 4:** Multivariate stepwise regression analysis showing the factors independently associated with OSTA in age and sex subgroups

	Characteristics	*β*	SE	Standardized *β*	*P*
Male population	Age	− 0.170	0.013	− 0.535	< 0.001
	WC	0.130	0.013	0.396	< 0.001
	baPWV	− 0.001	0.000	− 0.195	< 0.001
	Ccr	− 0.013	0.006	− 0.086	0.042
Female population	Age	− 0.187	0.005	− 0.612	< 0.001
	WC	0.035	0.007	0.130	< 0.001
	baPWV	− 0.001	0.000	− 0.190	< 0.001
	BMI	0.300	0.021	0.379	< 0.001
	DBP	0.011	0.005	0.038	0.033
Ages < 65population	baPWV	− 0.001	0.000	− 0.190	< 0.001
	BMI	0.400	0.018	0.540	< 0.001
	Sex	− 1.139	0.134	− 0.204	< 0.001
Ages 65+ population	baPWV	− 0.002	0.000	− 0.304	< 0.001
	WC	0.120	0.011	0.440	< 0.001
	Sex	− 2.169	0.193	− 0.451	< 0.001

Note: Multivariable stepwise regression analysis was performed using variables showing significant relationships with OSTA in Spearman's rank correlation analysis

*BMI* body mass index, *WC* waist circumstances, *Ccr* creatinine clearance, *baPWV* brachial-ankle pulse wave velocity, *OSTA* Osteoporosis Self-Assessment Tool for Asia, *DBP* diastolic blood pressure, *HDL* high-density lipoprotein cholesterol

## Discussion

The present study, conducted in a large Chinese community-based cohort, showed that baPWV, a promising yet relatively simple test, has a significant negative correlation with the degree of risk for osteoporosis as quantified by OSTA. The association between baPWV and osteoporosis risk was independent of age, sex, and traditional risk factors, which was concluded using the multivariable analysis. For predicting high risk of osteoporosis and fracture, we showed a baPWV cutoff value of 1693 cm/s that had the best predictive power resulted in AUCs of about 0.722.

Correlations between arterial stiffness and BMD have been frequently reported in both retrospective and cross-sectional studies. A cross-sectional study involving around four thousand Chinese men and women aged 65–92 reported that ankle-brachial index (ABI) as a measurement for peripheral arteriosclerosis was positively correlated with hip BMDs [38]. Further, prospective studies have also reported findings in evaluating whether low BMD predicts cardiovascular events. In China, a prospective osteoporosis study followed 1724 postmenopausal women for 5 years; they found the presence of aortic calcifications assessed using semiquantitative radiography at baseline was associated with a higher rate of vertebral fractures (12.2% vs. 4.5% in women with and without aortic calcifications (*P* = 0.01) [39]. This study was pooled in a further meta-analysis on the relationship of aortic calcifications to the risk of fracture, which demonstrated that aortic calcifications were significantly and independently associated with a higher fracture risk, recruiting 14,632 participants in total [38]. For peripheral arterial disease (PAD), in a prospective study of 1332 individuals, peripheral arterial disease (PAD) measured by ABI was not associated with the occurrence of fractures [40]. However, another study involved measurements of ABI and of BMD in 5781 men aged 65 years or older found inconsistent results with the previous mentioned study, which reported that individuals with PAD had higher rates of bone loss and increased risk of non-spine fractures [41].

The possible pathophysiological mechanism linking high baPWV and osteoporosis could be oxidative stress, an imbalance between exposure to toxic reactive oxygen species (ROS) and antioxidant systems, considering that it is associated with both arterial stiffness and osteoporosis [42, 43]. Hormonal changes associated with menopause and aging affect both arterial stiffness and bone resorption and reconstruction; it could be another mechanism behind the predictive value of baPWV for osteoporosis risk [44]. Diverse studies revealed significant correlations between the severity of arterial stiffness/osteoporosis and inflammatory markers; it could also be another common pathway for the pathogenesis of arterial stiffness and osteoporosis [45]. Since in population ages 40 to 65, we found the highest predictive value of baPWV in individuals at high risk of osteoporosis, proposing arterial stiffness and osteoporosis might co-progress from the early stages of bone loss through common mechanisms.

**Fig. 3:**
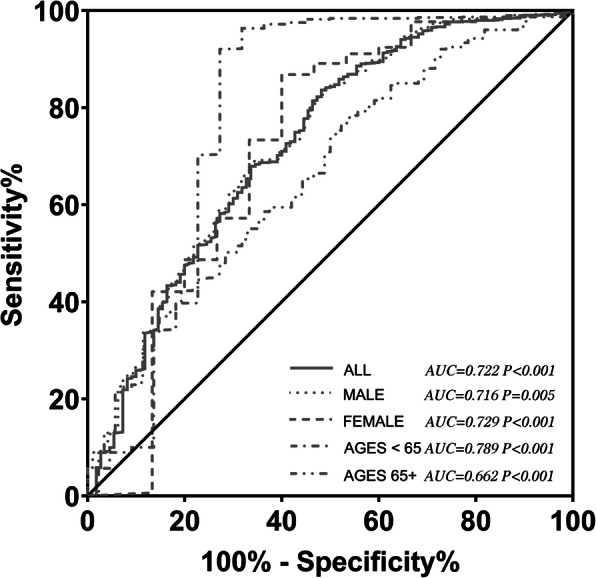
ROC curve of the association between baPWV and the high risk of osteoporosis and fracture (corresponding to OSTA < − 4.0) in total sample and subgroups. ROC, receiver operating characteristic; baPWV, brachial-ankle pulse wave velocity; OSTA: Osteoporosis Self-Assessment Tool for Asia; AUC, area under curve; CI, confidence interval

It is important to obtain a simple and effective way to predict osteoporosis and cardiovascular disease risk, considering the huge aging population of the People’s Republic of China. As far as we know, the first study to report the relationship between OSTA and baPWV in Chinese population was published in 2019, involving 129 elderly individuals and suggests the OSTA index was negatively correlated with baPWV in linear regression analysis and baPWV as a valuable predictive factor for potential osteoporotic risk [46]. Our findings have proven the association in a large Chinese community-based cohort involving 1407 individuals; the AUC was 0.722 in ROC curve analysis for evaluating baPWV as a predictor of high risk of osteoporosis and fracture (OSTA ≤ − 4), close to the AUC (0.742) reported in the abovementioned study [46]. However, we reported a low sensitivity value of 51.8% for the optimal cutoff point of baPWV value in predicting the presence of high risk of osteoporosis and fracture. This is probably due to the age heterogeneity in our study, since we recruited all the adults aged 40 years and older (Fig. 3).

Low BMI having been frequently shown to increase risk of fracture, possibly due to its association with bone loss, less soft tissue, and muscle weakness [47]. In our study, we found BMI and WC are positively associated with OSTA. However, there were studies reporting high BMI increases the risk of osteoporotic and hip fractures risk [48]. In a meta-analysis involving 398,610 women, it is interesting to find that the association between BMI and fracture risk is complex, which differs across skeletal sites. Since there is no consistent conclusion from these analyses, a healthy and normal BMI might be suggested to help minimize the risk of fracture risk. Only few observational studies have investigated the association between serum lipid level and bone fractures in Chinese population, and the conclusions between studies are also controversial. In our study, we found a negative association between HDL and OSTA. In a cross-sectional study involving around 5000 healthy volunteers, the authors reported that the subjects with a BMI lower than18.5 had a higher incidence of osteoporosis than BMI ≥ 18.5 in both sexes [49]. Another cross-sectional study including 1791 participants (62.1% postmenopausal women and 213 fractures) reported a significant positive association between HDL-C level and risk of osteoporotic fracture in total participants (OR 1.37, *P* = 0.023). These findings are consistent with what have been reported in our study. However, a recent study applied two-sample Mendelian randomization (MR) methods to explore the causal association between blood lipid levels and fracture. They reported that HDL may have an indirect influence on fracture mediated by BMD [50]. According to what we have discussed above, the relationship between lipid levels in the blood and the risk of fracture is currently controversial and the causal association remains elusive; further research is required.

The study described here included 1407 participants aged 40 years and older. This relatively large sample size and subgroup analysis strengthens the thoroughness of our findings. According to what we have found, we proposed that baPWV had moderate discrimination ability for high risk of osteoporosis, especially for population ages 40 to 65. However, several limitations of our study should be thoroughly discussed. First, as a cross-sectional study, no causal inference can be concluded. Further well-designed longitudinal studies are needed to validate the relationship identified in this study. Second, OSTA was the only measurement representing osteoporosis and fracture risk in this study. Future studies should search a link between other markers of osteoporosis and arterial stiffness, such as Mandibular cortical width, a marker of osteoporosis detected by dental panoramic radiographs [51]. Third, information such as physical activity and alcohol consumption was not collected. These behavioral factors might have to affect fracture risk profoundly.

## Conclusion

In summary, we have found the independent predictive value of baPWV for osteoporosis risk in a large Chinese community-based cohort. Furthermore, the inverse association of the OSTA index and baPWV was statistically significant. baPWV may be a simple and useful indicator of osteoporosis and fracture risk.

## Supplementary Information

**Additional file 1: Table S1.** Youdem index for the ROC curve analysis.

## Data Availability

The datasets used and/or analyzed during the current study are available from the corresponding author on reasonable request.

## Abbreviations

baPWV: Brachial-ankle pulse wave velocity
OSTA: Osteoporosis Self-Assessment Tool for Asia
BMD: Bone mineral density
ROC: Receiver operating characteristic
AUC: Area under the curve
SD: Standard deviation
PAD: Peripheral arterial disease
ROS: Reactive oxygen species
BMI: Body mass index
WC: Waist circumstances
SBP: Systolic blood pressure
DBP: Diastolic blood pressure
FPG: Fasting plasma glucose
PPG: Postprandial glucose
HDL: High-density lipoprotein cholesterol
LDL: Low-density lipoprotein cholesterol
TC: Total cholesterol
TG: Triglyceride
Ccr: Creatinine clearance
